# The Laminar Cortex Model: A New Continuum Cortex Model Incorporating Laminar Architecture

**DOI:** 10.1371/journal.pcbi.1002733

**Published:** 2012-10-18

**Authors:** Jiaxin Du, Viktor Vegh, David C. Reutens

**Affiliations:** The University of Queensland, Centre for Advanced Imaging, Brisbane, Queensland, Australia; Université Paris Descartes, Centre National de la Recherche Scientifique, France

## Abstract

Local field potentials (LFPs) are widely used to study the function of local networks in the brain. They are also closely correlated with the blood-oxygen-level-dependent signal, the predominant contrast mechanism in functional magnetic resonance imaging. We developed a new laminar cortex model (LCM) to simulate the amplitude and frequency of LFPs. Our model combines the laminar architecture of the cerebral cortex and multiple continuum models to simulate the collective activity of cortical neurons. The five cortical layers (layer I, II/III, IV, V, and VI) are simulated as separate continuum models between which there are synaptic connections. The LCM was used to simulate the dynamics of the visual cortex under different conditions of visual stimulation. LFPs are reported for two kinds of visual stimulation: general visual stimulation and intermittent light stimulation. The power spectra of LFPs were calculated and compared with existing empirical data. The LCM was able to produce spontaneous LFPs exhibiting frequency-inverse (1/ƒ) power spectrum behaviour. Laminar profiles of current source density showed similarities to experimental data. General stimulation enhanced the oscillation of LFPs corresponding to gamma frequencies. During simulated intermittent light stimulation, the LCM captured the fundamental as well as high order harmonics as previously reported. The power spectrum expected with a reduction in layer IV neurons, often observed with focal cortical dysplasias associated with epilepsy was also simulated.

## Author Summary

Local field potentials (LFPs) are low-frequency fluctuations of the electric fields produced by the brain. They have been widely studied to understand brain function and activity. LFPs reflect the activity of neurons within a few square millimeters of the cerebral cortex, an area containing more than 10,000 neurons. To avoid the complexity of simulating such a large number of individual neurons, the continuum cortex model was devised to simulate the collective activity of groups of neurons generating cortical LFPs. However, the continuum cortex model assumes that the cortex is two-dimensional and does not take into account the laminar architecture of the cerebral cortex. We developed a three-dimensional laminar cortex model (LCM) by combining laminar architecture with the continuum cortex model. This expansion enables the LCM to simulate the detailed three-dimensional distribution of the LFP within the cortex. We used the LCM to simulate LFPs within the visual cortex under different conditions of visual stimulation. The LCM reproduced the key features of LFPs observed in electrophysiological experiments. We conclude that the LCM is a potentially useful tool to investigate the underlying mechanism of LFPs.

## Introduction

Neuronal activity changes the distribution of electric potentials in the brain [1], [2]. Local field potentials (LFPs) are the low-frequency (<100 Hz) fluctuations in electric potentials in the extracellular space of the brain [2], [3]. They represent a weighted average of the potential changes produced by neuronal activity in a small volume around the measuring electrode [4], [5]. Concurrent electrophysiological and functional MRI experiments have also demonstrated that LFPs are correlated with signal change in functional magnetic resonance imaging, a method of detecting neuronal activity through changes in blood-oxygen-level-dependent signal [6]–[8]. Previous electrophysiological experiments investigating the neuronal processes underlying LFPs have measured the membrane potential of neurons and extracellular field potentials simultaneously [9], [10]. A major difficulty with this paradigm is that LFPs reflect the activity of more than 10,000 neurons [11] within 250 micrometres of the recording electrode [4], [5]. Simultaneous measurement of such a large number of neuron activities has not been achieved to date. Furthermore, multiple concurrent processes contribute to LFPs, including action potentials, synaptic transmission, glial activity, and even extracellular space diffusion [12] and are difficult to disambiguate.

Computer simulations have widely been adopted to predict changes in neuronal activity associated with corresponding LFPs. A previous study simulated the membrane potential changes of a large number of individual neurons as means of reconstructing the LFP [for example, see 13]. Simulating the dynamics of a large number of neurons faces the challenge of specifying the physiological parameters in large, inhomogeneous populations with diverse physiological properties [14]. An alternative approach to simulating individual neuronal activity has been to simulate the activity in an ensemble of neurons. An example of this is the continuum cortex model, developed by Wright *et al* [15]–[18], which has been used to simulate ensemble activity at different scales [17]. Existing continuum cortex models do not take into account the laminar architecture of the cerebral cortex. They are, therefore, limited in their ability to model the distribution of electric potential of the brain in three dimensions. Cortical neurons are organized in columns comprising as many as 20,000 neurons [19], [20]. Functionally, neurons in a column display similar responses to specific stimuli [21]. In this paper, we build on this notion to expand the continuum cortex model by incorporating the laminar connection architecture of the cortex and simulating the collective of neuronal ensembles within cortical columns. We have used the new laminar cortex model (LCM) to simulate LFPs within the visual cortex under different conditions of visual stimulation.

## Methods

### Continuum cortex model

We give a brief overview of the continuum cortex model for completeness, but for specific details refer to [17]. The continuum cortex model simulates the collective electrophysiological activities of the cerebral cortex. A population approximation is used to overcome the difficulty of simulating a large number of individual neurons, and to capture the essential aspects of cortical dynamics [15], [16]. The continuum cortex model divides the simulated cortical area into a 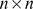 grid of elements, where 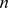 is an integer. Each element consists of two populations of neurons: excitatory and inhibitory [17]. Each population is treated as a single entity capable of receiving spikes, changing membrane potential, and generating and propagating spikes [17].

The numbers of spikes propagating between neurons of two groups at any one time varies. In the continuum cortex model the effects of action potential shape and its temporal evolution are ignored. Instead, the average afferent spike rate (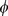) is used to measure interaction between the two groups of neurons. The spike rate is defined as the average number of spikes a neuron of one group receives from a neuron of the other group per unit time.

The continuum cortex model contains four main components: 1) spike generation, 2) spike propagation, 3) generation of the postsynaptic potential, and 4) membrane potential aggregation. The equations describing each component are provided in Text S1 and were developed either by using theoretical approaches or by experimentally fitting observed data using an appropriate function. The mean field approximation was employed during this procedure [17].

### Cortical laminar connection

**Figure 1:**
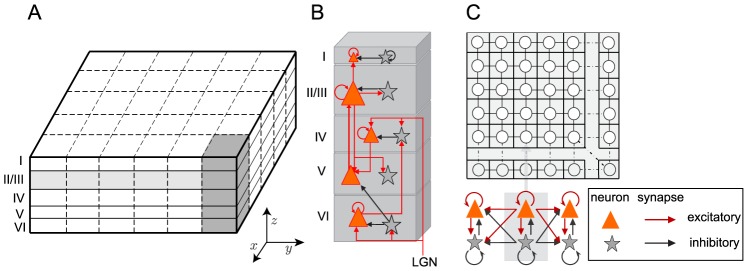
The configuration of the LCM. (A) The LCM simulates five cortical layers. Cortical layers are discretized to a grid of elements, which contain two neuron groups: excitatory and inhibitory. (B) The laminar connection between cortical layers is illustrated. Only the strong connections are shown in the figure. For the complete connection map please refer to Table S1 of Text S2. (C) The connections between neuron groups within a lamina are shown.

The LCM exploits the laminar architecture of the cortex. Five cortical layers (layer I to VI) are considered (cortical layers II and III are combined). Each layer is simulated using the continuum cortex model, and the layers are connected by laminar synaptic connections (see Figure 1). A synaptic connection map is created and used to control the connection between and within cortical layers (see Table S1 in Text S2). This connection map was based on empirical observations of the number of synapses formed between different types of neurons by Binzegger [22] (see Text S2). The connection map classifies the afferent synapses on each group of cortical neurons into three categories: 1) intracortical synapses, from within the visual cortex (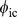), 2) cortico-cortical synapses, from other cortical areas (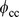), and 3) thalamic synapses, projections from neurons in the lateral geniculate nucleus (LGN, 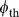).

The LCM allows simulation of centimeter and column scale (micrometer) cortical regions [17]. Since the grid elements of the centimeter scale model correspond to the size of cortical columns, the connections between cortical laminae are assumed to be local. This means that elements in the same horizontal position of all cortical layers are connected vertically (see Figure 1B). In contrast, the column scale implementation is approximately the size of one cortical column. Therefore, connections between cortical layers are global, and the average spike rate of a cortical layer is the input to other cortical layers. The work here is focused on simulating LFPs produced in the visual cortex. Hence, results are limited to the application of the centimeter scale model.

### Visual stimulus

**Figure 2:**
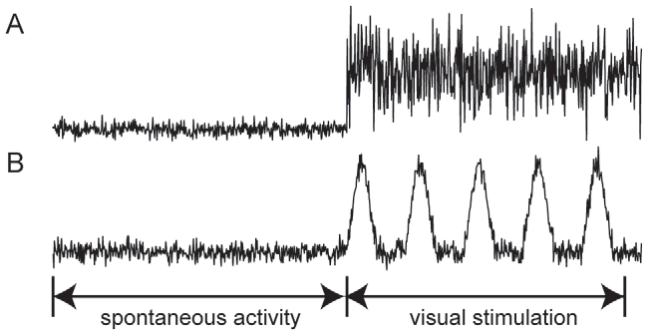
Afferent spike rates corresponding to visual stimulations. (A) Spike rates correspond to spontaneous activity followed by constant visual stimulation, and (B) spike rates represent to spontaneous activity prior to intermittent light stimulation.

We simulated the effect of visual stimulation on LFPs using the LCM. Different forms of visual stimulation were assumed to form different spike trains projecting from the LGN to deeper cortical layers of the visual cortex (Layer IV, V and VI, see Table S1 in Text S2). Three states of visual stimulation were examined in the model: 1) spontaneous activity without visual stimulation, 2) constant visual stimulation, and 3) intermittent light stimulation. As illustrated in Figure 2, these conditions correspond to afferent spike trains with the shape of small amplitude white noise, large amplitude white noise (the random number generator from [23] was adopted), and recurring Gaussian peaks, respectively.

Apart from the synapses projecting from neurons in LGN and the visual cortex, there are also a large number of synapses originating from other cortical areas (see Table S1 in Text S2). We assume that spikes from these synapses contribute to background noise, which was modeled as low-amplitude white noise.

### Model parameters

The LCM has over 150 parameters, which fall into four categories relating to: 1) electrophysiological properties of neurons, 2) spike propagation, 3) synaptic transmission, and 4) connections between cortical laminae. Most of these parameters were estimated from experimental data, while others were left as free parameters. However, the cortex is complex, to the extent that our simplified parameters may not represent its physiology, morphology and architecture exactly. We found that a small deviation of the parameter values do not change the results reported here significantly. This is because a similar LFP outcome can be achieved by tuning free parameters.

Parameters relating to the electrophysiological properties of neurons are well established in the literature. We used the same values, derived from experimental data, as the continuum cortex model [17].

Spike propagation parameters and their values used are listed in Table 1. The propagation speed of spikes in the horizontal (lateral) direction (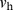) was set to 0.24 m/s, which is consistent with experimental measurements of the speed of spread of spikes in the cortex [24], [25]. Since collateral branches are usually smaller in diameter than the main axon, the speed of vertical (inter-laminar) propagation of spikes (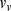) was set to 1.2 times the speed of horizontal propagation. The spike propagation range parameters were set to the similar values as continuum cortex model [17].

**Table 1:** Spike propagation parameters.

Parameter	Representing	Value
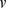	Spike propagation speed	Horizontal: 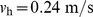
		Vertical: 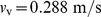
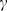	Spike propagation range	Excitatory: 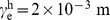
		Inhibitory: 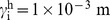

There is a wide range of published values for synaptic transmission parameters [26], [27]. We chose the middle parameter value when a range was provided and the average when multiple values were reported. The excitatory and inhibitory synaptic gains 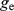 and 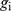, were treated as free parameters. Their values were determined by fitting experimental data to the LFPs generated using the LCM.

The best set of parameter values was selected as those fulfilling the following criteria: 1) the LFP power spectrum fitted the 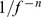 function with 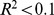 [28]. 2) with simulated visual stimulation, there was an increase in gamma frequency in the power spectrum; 3) membrane potentials of neuron groups were less than 10 mV above their resting membrane potentials [29].

### Simulation

The simulation program was written using the ANSI C language and compiled with the Intel C compiler (http://software.intel.com/intel-compilers/). The program was compiled and executed on a Linux workstation (Dell® Precision T7500) with Ubuntu version10.10 (×86_64, http://www.ubuntu.com). OpenMP (http://www.openmp.org), a shared-memory parallel programming library, was used to parallelize the code to speed up program execution.

In this paper, the LCM was used to simulate a cortical area of size 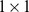 cm^2^. The domain was discretized to a 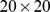 grid. At the beginning of each execution of the program, the simulation time was initialized to zero, and every neuron state variable was set to its resting state value (see Text S2). The iteration time step was one millisecond. After initialization, the program executed without particular visual stimulation for 60 seconds at which time the system is assumed to have reached steady state. Constant visual stimulation or intermittent light stimulation was then applied for 20 seconds (time = 60–80 sec). LFPs were simulated for conditions of spontaneous activity and for each mode of visual stimulation.

### Data analysis

In the simulation, the membrane potentials of all neuron groups in the middle element of a layer are recorded during the entire execution. Data of the last 1.024 second prior to visual stimulation and after stimulation were used for frequency spectrum analysis.

For comparisons with experimental data, the LFPs of the simulated cortical area are assumed to be the average of neuronal membrane potentials of the central elements of all layers, stated as:
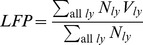
(1)where 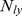 are the numbers of neurons in the central element of layer 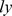 and 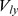 are the potentials of the central elements of layer 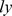, which is the average of membrane potentials of neurons in the element, that is
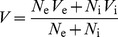
(2)where 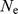, 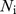 are the numbers of excitatory and inhibitory neurons and 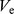 and 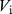 are the (average) membrane potentials of excitatory and inhibitory neuron populations respectively. The frequency spectrum of the LFPs was computed using the *fast Fourier transform* as implemented in MATLAB 2010a (http://www.mathworks.com). The LFP frequency power spectra were compared with experimentally measured data.

LFPs produced by LCM were also used to estimate current source density. The standard one-dimension current source density calculation method was used [30], [31]

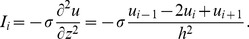
(3)Here 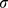 is electric conductance of the cortex, and was set to 0.3 S/m, 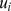 is the potential at the 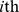 point, and 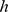 is the distance between two adjacent points. To reduce spatial noise, the three-point Hamming filter was applied [32], [33]

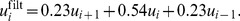
(4)


## Results

### Parameter sensitivities

We examined the behaviour of the LCM using different parameter values. For each parameter combination, around 100 executions of the LCM were conducted, and the average LFP frequency spectrum was computed.

**Figure 3:**
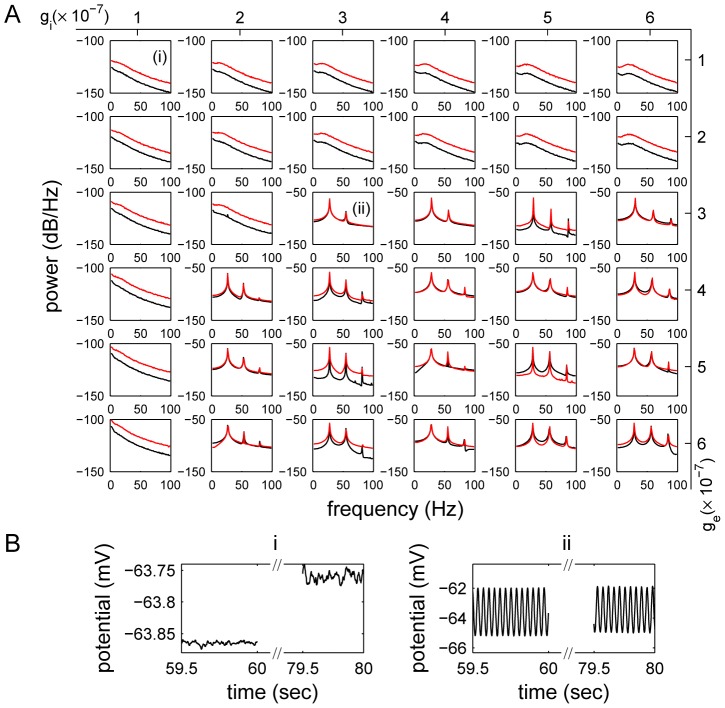
The effect of changing synaptic gains on the LFP power spectra. (A) LFP power spectra were obtained using LCM with different combinations of excitatory (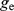) and inhibitory (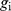) synaptic gains. Black lines show the power spectra of spontaneous LFPs and red lines correspond to the activated LFPs. A more detailed synaptic gain dependent frequency map is provided in Figure S1. (B) The time serials of LFPs obtained in one run with two synaptic gain combinations (i) 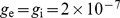 V/spike, and (ii) 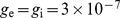 V/spike, as corresponding to sub-figures (i) and (ii) in (A).

Figure 3 shows the power spectra of the LFPs obtained with different synaptic gains. The LCM was able to generate LFPs with different types and envelopes of oscillation, depending on the combination of excitatory and inhibitory synaptic gains used in the simulation. For example, when either excitatory or inhibitory synaptic gain was small, the frequency spectrum of background activity had an inverse-frequency shape. Stimulation resulted in an increase in gamma frequency. In contrast, when the excitatory and inhibitory synaptic gains were both large, particular frequency peaks dominated the LFP power spectra. Thus, variations of synaptic gains had a strong impact on LFP frequencies.

For large synaptic gains, the peaks in the power spectra did not change position with variation in synaptic gain. Dependence of peak position on other parameters was also examined by generating LFP power spectra with different parameter values. The time course of the postsynaptic potential (PSP) was found to be strongly correlated with the positions of the peaks. Peak frequency decreased with increasing PSP time course. (Four examples of LFP power spectra with different PSP time courses are shown in Figure S2). This suggests that the dominant oscillation frequency is controlled by the feedback between excitatory and inhibitory neurons.

The shape of the power spectrum of LFPs generated by the LCM is controlled by the balance between excitatory and inhibitory postsynaptic potentials (PSPs). These are influenced by many parameters simultaneously, including synaptic gains, spike propagation ranges and synapse numbers. Changes in PSPs caused by variation of one parameter could be compensated by other parameters. For example, increase of synaptic gains may not change PSP when the corresponding synapse number is decreased. Therefore, the LCM could produce similar LFPs using different combinations of parameter values.

**Figure 4:**
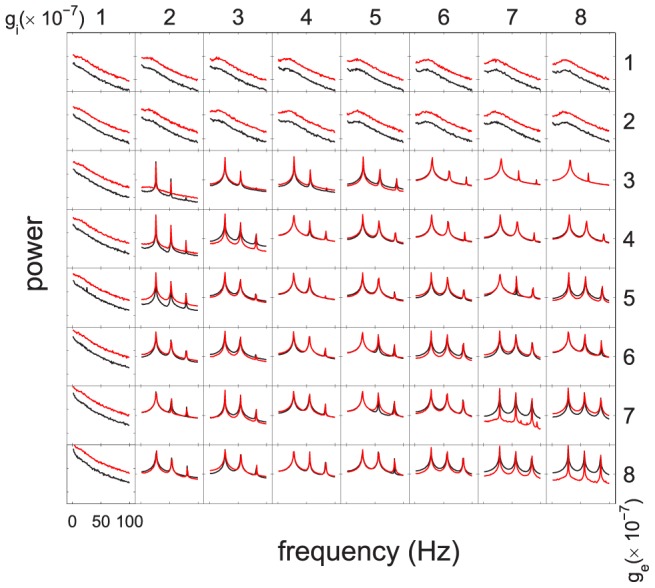
The effect of changing cortical architecture on LFP power spectrum. This figure shows power spectra produced by LCM configured with different synaptic gains, and presynaptic neurons in layer IV decreased by 50%. The red lines and black lines illustrate the power spectra of activated and spontaneous LFPs.

Experimental models of neocortical epileptic foci suggest that reduced synaptic inhibition in layer IV plays an important role in epileptogenesis [34], [35]. Focal cortical dysplasias characterized by an absence or significant reduction in layer IV are also very frequently associated with epilepsy [36]. Figure 4 shows the LFP power spectrum shapes generated by the LCM when the numbers of synapses formed with presynaptic neurons in layer IV are decreased by 50%. Compared to Figure 3A, the power spectra show a small shift to small inhibitory gain. For example, for LFPs produced using excitatory and inhibitory synaptic gains of 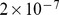 V/spike, the power spectrum changed from a frequency-inverse 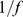 shape to one with spectral peaks as would be expected with seizures when presynaptic neurons of layer IV decrease by 50%. This suggests that, changes in neuron or synapse density may change the way LFPs oscillate dramatically. These alterations in dynamics may increase our understanding of how abnormalities in cortical architecture lead to seizures.

### Spontaneous and visually stimulated local field potentials

**Figure 5:**
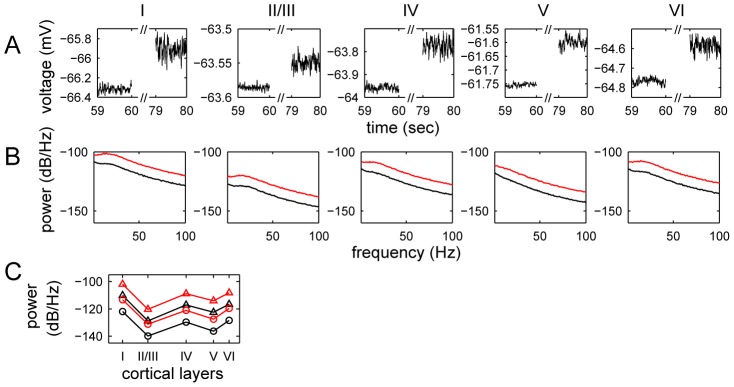
The temporal variations and power spectrum of membrane potentials in cortical layers. Illustrated are (A) simulated field potentials of layer I, II/III, IV, V and VI, and (B) their corresponding power spectra for the general visual stimulation experiment, and (C) the average power spectra of LFPs in the gamma frequency (30–100 Hz, circles) and sub-gamma frequency (5–20 Hz, triangles) during spontaneous activity (black lines) and general stimulation (red lines). In (B) the black lines depict the resting state LFPs and red lines show the outcome of stimulation. The data are obtained using 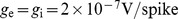.

Figure 5 shows the time courses of membrane potentials in a single run of the LCM. We found that in every cortical layer, membrane potentials oscillated with amplitudes of 0.05–0.2 mV; the amplitudes are much larger in layers IV and VI (around 0.1 mV) than in other layers (around 0.05 mV). During stimulation, the membrane potentials and its oscillation amplitudes increased in all layers except layer I. The power spectra in all layers, as provided in Figure 5, all showed inverse-square decreasing frequency background activities, which is observed experimentally [37]. Stimulation also increased high-frequency membrane potential oscillation of all deep layers.

**Figure 6:**
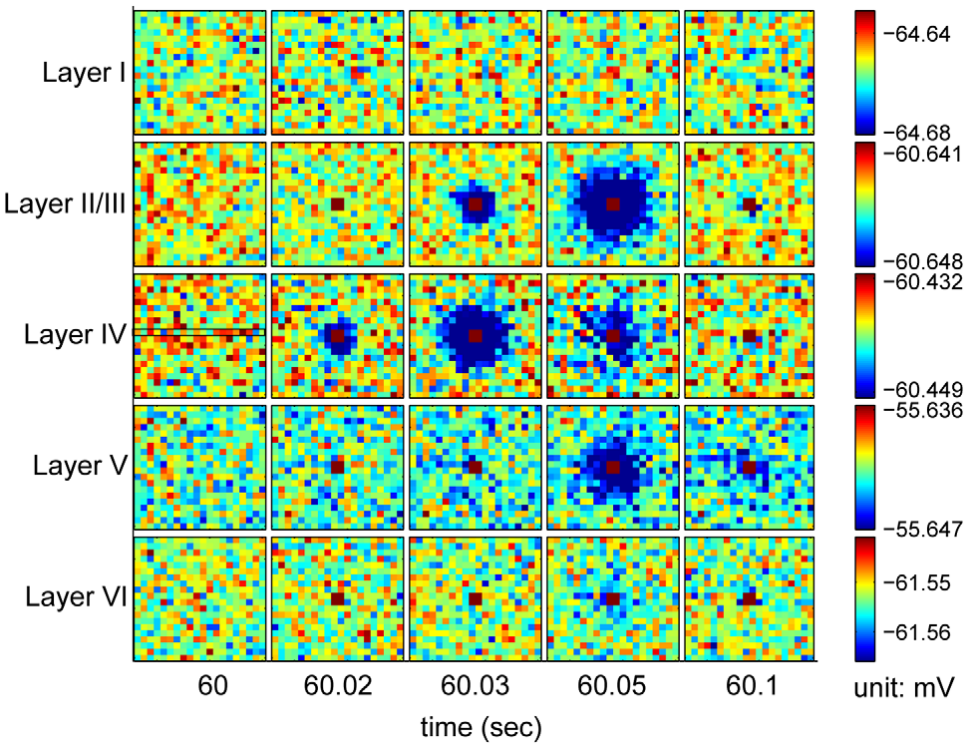
Potentials in the cortex driven by a single transient source. The four central elements in layer IV are driven by 100 spike/sec LGN input starting after 60 seconds of spontaneous activity. The spike source lasts for 20 milliseconds. The following parameters were used: 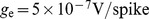, 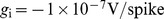, 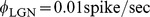 for spontaneous activity and 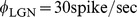 for a spike source in the central four elements of layer IV.

**Figure 7:**
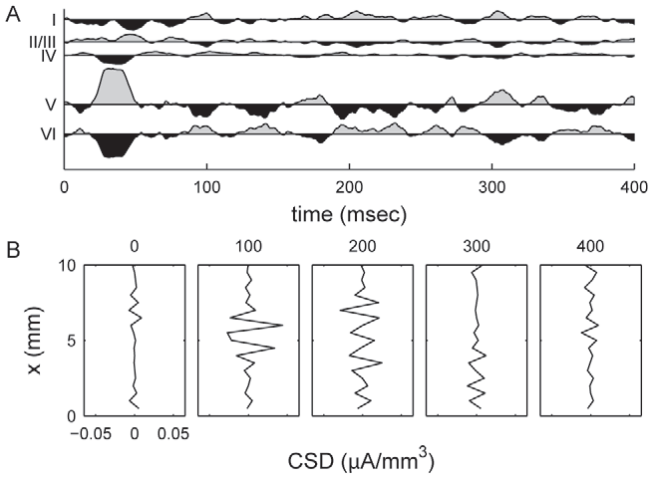
Current source densities (CSD) generated by the LCM. Shown are (A) CSDs for the central elements of each cortical layer, and (B) temporal profile for current source density of the central line of layer IV (see Figure 6). The CSD plots show the difference between CSD at each time point and the mean value in the entire epoch. Time values are in milliseconds after the onset of transient LGN input. A positive CSD value indicates a current source. Results are calculated from the same dataset as Figure 6.

The laminar distribution of the LFP power spectrum amplitude was examined. Figure 5C shows the laminar distribution of the average of the LFP power distribution in the gamma frequency (30–100 Hz) and sub-gamma frequency (5–20 Hz) ranges for spontaneous activity and general stimulation. Higher frequency powers were observed in layers IV and VI. This is in agreement with experimentally measured laminar LFP amplitude profiles in the primary visual cortex [38]. Since layers IV and VI are the main layers of the visual cortex receiving and sending projections to the LGN, the observed variation in LFP power spectra amplitudes between layers most likely results from these projections. We simulated the propagation of one spike source in the cortex using LCM. In Figure 6 we provide the result when a spike source is placed in the four central elements of layer IV for 20 milliseconds after 60 seconds of spontaneous activity. Following spike onset, a strong potential is observed in the center of all cortical layers except layer I. The potential is decreased in elements surrounding the source, simulating surround inhibition. We display the temporal profiles of current source density along a transverse line through the central point in layer IV and for the central elements of each cortical area in Figure 7.

### Steady-state visual evoked potentials (SSVEPs)

Many electrophysiological experiments have demonstrated that with intermittent light stimulation, neuronal activity in the visual cortex synchronizes with stimulus frequency [39]–[42]. Furthermore, EEG responses are enhanced at this frequency (fundamental harmonics), as well as at half the stimulus frequency (first sub-harmonic), and at multiples of the stimulus frequency (multiple harmonics). The responses to visual stimulation at specific frequencies, termed steady-state visual evoked potentials (SSVEPs), can be observed on both scalp EEG recordings [39] and invasive recordings of LFPs [40]. We used SSVEPs to examine the effect of cortical architecture on LFPs.

**Figure 8:**
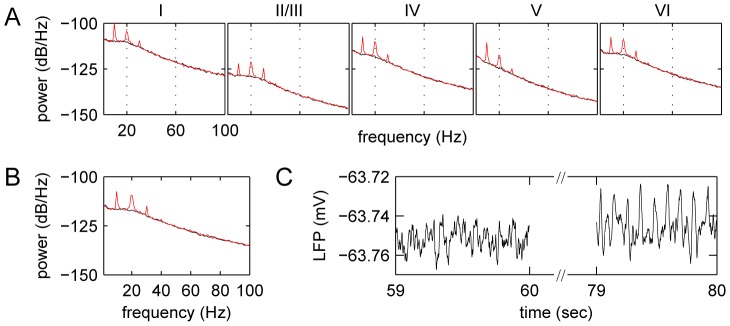
Power spectra of membrane potentials for SSVEPs generated with the LCM. The figure shows (A) the power spectra of membrane potential in layers I, II/III, IV, V, VI and (B) power spectra of the LFP produced by the LCM under intermittent light stimulation. The black lines show power spectra of spontaneous LFPs, and red lines illustrate stimulated LFP power spectra. In (C) an example of LFPs before and after intermittent light stimulation in a single run is also shown. The following parameters were used: 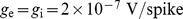, 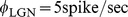 for spontaneous activity.

The LCM was used to simulate LFPs with 10 Hz intermittent light stimulation represented by a Gaussian distribution of spike rates for neurons projecting from the LGN to the visual cortex. The peak and standard deviation of the Gaussian shape was 30 spikes/second and 6.25 milliseconds, respectively (see Figure 2). Figure 8 shows the variation of LFPs with time and the associated power spectra. Simulations using the LCM reproduced the power spectra reported in experimental data [39]. The LFP power spectrum had peaks at frequencies that were multiples of the stimulus frequency (i.e. capturing multiple harmonics). Notably, the amplitude of fundamental harmonic (i.e. frequency peak at 10 Hz) was smaller in layer II/III than other layers. This is probably because there are fewer projections from LGN to layer II/III than other layers. Experimentally observed sub-harmonics were not obvious in simulations using the LCM [39].

## Discussion

This paper introduces the LCM and describes its use to simulate LFPs in the primary visual cortex. The LCM has the advantage that it incorporates the architecture of the visual cortex allowing the simulation of LFPs with high spatial and temporal resolution. We were able to simulate the membrane potential in each cortical layer, as well as its temporal variations. We used the LCM to investigate the relationship between visual stimulation and LFPs. We validated the model using two different experimental simulations: constant visual stimulation and intermittent light stimulation. Our results were comparable to relevant experimental measurements. We also simulated the effects of changes in neuronal density in layer IV, often observed in epileptic cortical dysplastic tissue. For certain parameter combinations the changes in the power spectra were those expected in seizures. CSD maps showed comparable features to experimental data and intralaminar CSD profiles following transient LGN input had the appearance of surround inhibition.

With constant visual stimulation, the LCM produced LFPs oscillating in two different ways determined by the combination of parameters used in the simulation. When the cortex was activated with low levels of background noise and stimulus input (small synaptic gains), the LFP oscillation was governed by the pool of excitatory neurons. Synaptic transmission acts as a filter due to the convolution in the membrane potential aggregation function of LCM (refer to Equation S1.8 in Text S1). Effectively, this dampens high frequency oscillations and results in an inverse-squared decreasing LFP spectrum. However, when the cortex is highly activated, inhibitory neurons play a more dominant role, resulting in oscillations in which initial activation of inhibitory neurons leads to suppression of the membrane potential of all neurons, including the inhibitory pool followed by a burst of activity cause by excitatory input.

The LFPs produced using low synaptic gains are comparable to experimentally observed LFPs in the normal brain, while the LFPs obtained with large synaptic gains are similar to those measured during seizures [37]. This suggests that changes in neuronal physiology can result in a change in the LFP power spectrum and may help to explain frequency changes in the EEG observed in certain neurological disorders. There are some differences between LFPs from the LCM and experimentally measured LFPs. The amplitude of low frequency (<10 Hz) LFPs produced by the model is lower than measured experimentally. A possible explanation is that the low frequency oscillation results from feedback loops between the visual cortex and other brain areas [43], which are not considered in the LCM. The gamma frequency (40–200 Hz) power of stimulated LFPs is also smaller than experimental measurements. We postulate that this is because extracellular potential changes caused by synaptic activities and spike conduction are not included in the calculation of LFPs. These are reported to have a greater influence on high frequency LFPs [44]–[46]. The LCM simplifies synaptic processes and spike propagation to a signal delivery level. It does not simulate the burst of synaptic transmission and spikes.

The CSDs calculated from LCM recreates several features from experimental observations [47]. Within layers, the CSD profile simulated surround inhibition [48]. Across cortical layers, the temporal profile of CSDs was similar to those observed by Schroeder et al. [47] with transition from sink to source following stimulation.

We used SSVEPs, to test the effects of incorporating cortical architecture on simulation output. In our intermittent light stimulation study, we used the LCM to reproduce the behaviour of SSVEPs. The fundamental and high order harmonics were apparent in the visual cortex. The first sub-harmonics, shown to be present empirically [39], may be brought about by feedback loops between the primary visual cortex and other visual cortical areas. These connections are not included in the LCM.

The LCM may be used to simulate abnormal responses to intermittent light stimulation such as the photoparoxysmal response observed in forms of genetic generalized epilepsy. This can be achieved by varying LCM parameters, and comparing the simulation output with measured EEG data. This has the potential to generate testable hypotheses relating to underlying neurophysiological mechanisms.

Although we showed that LCM is able to reproduce some of the results of electrophysiological experiments, it has some limitations. Firstly, only two populations of neurons (excitatory and inhibitory) are considered. The behaviour of excitatory neurons may not be best captured by a single category. For example, fast-spiking neurons generate spikes differently from other excitatory neurons [27]. In future work we will extend the LCM to include multiple categories of excitatory neurons. Secondly, simulation of neurotransmission in the LCM may be oversimplified. For example, in its current form it cannot simulate the effects of activating fast (AMPA) and slow (NMDA) excitatory glutamatergic receptors on LFPs. Thirdly, the physiological parameters used in our simulation were obtained from the results of experiments conducted in different species. In our simulations, LFPs were calculated as the aggregate membrane potential dynamics of populations of neurons, an approach commonly employed in simulation studies e.g. [49]. This approach may be inaccurate because it does not take into account the filtering properties of the neural membrane [44], [45]. Methods based, for example, upon summation of conductance of synapses to pyramidal neurons [2], [45], [50] are inapplicable to the LCM, which simulates the collective activity of neuron groups. A future hybrid model is required to link continuum cortical models and models based on simulating the properties of individual neurons.

## Supporting Information

Figure S1 **Detailed map of synaptic gain dependent LFP frequency spectra.** Provided is the detailed map of LFP frequency spectra produced by LCM using different excitatory and inhibitory gains. The red lines show the frequency spectra of stimulated LFPs, while the black lines depict that of spontaneous LFPs.
(TIF)

Figure S2
**The shift of frequency peaks with different PSP time courses.** Provided are LCM produced LFP frequency spectra while the peaks of EPSP time courses are (A) doubled and (B) decreased by half, and the peak of IPSP time course is (C) doubled and (D) decreased by half. The following parameter values were used: 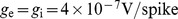.(TIF)

Text S1 **The state equations of the laminar cortex model.**
(DOC)

Text S2 **The meanings and values of the parameters used in the laminar cortex model.**
(DOC)
