# Supplementary figures and images for: The Laminar Cortex Model: A New Continuum Cortex Model Incorporating Laminar Architecture
Source: PLoS Comput Biol. 2012 Oct 18;8(10):e1002733. doi: 10.1371/journal.pcbi.1002733 (PMC3475685; doi:10.1371/journal.pcbi.1002733)

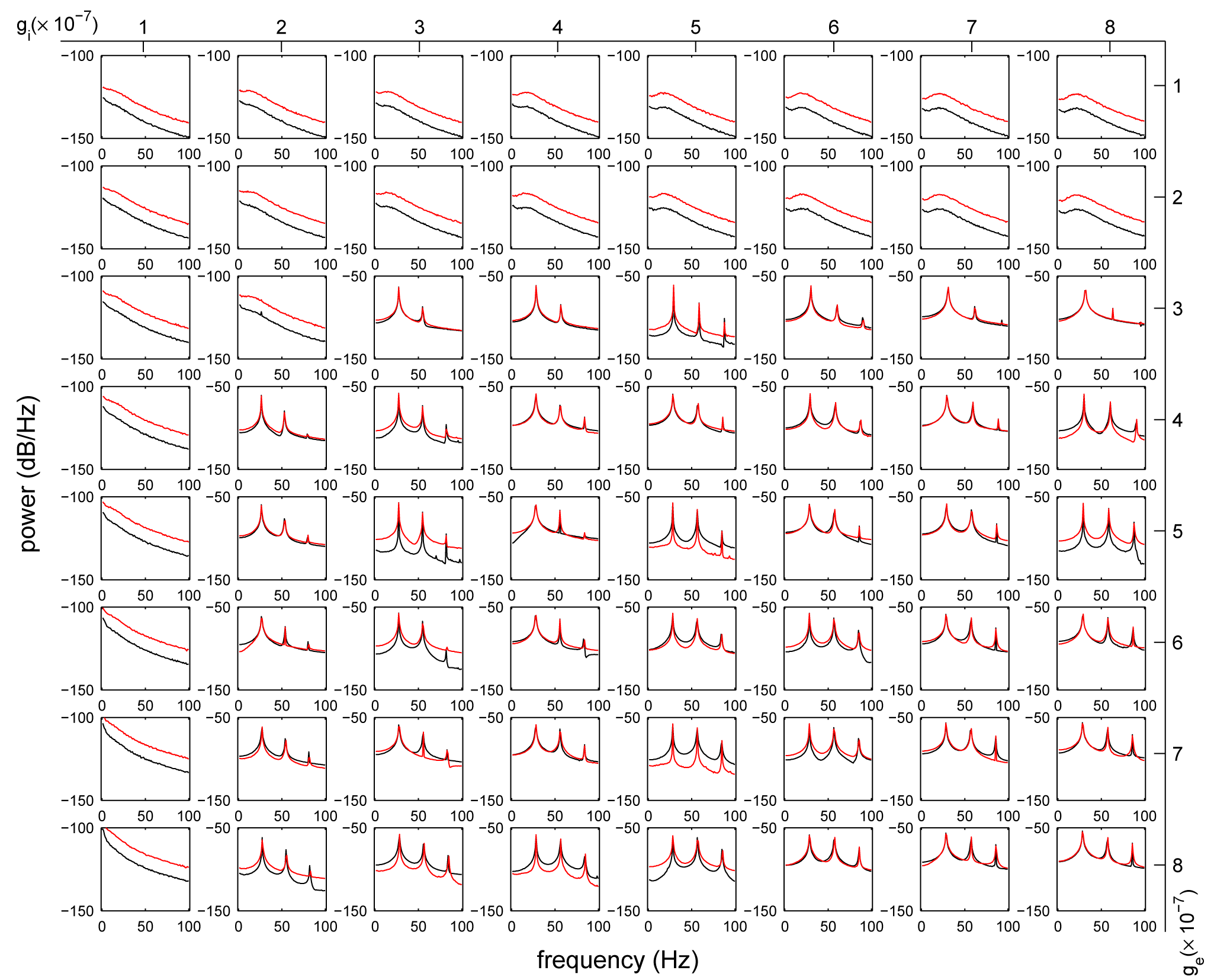

Supplement: Figure S1 — Detailed map of synaptic gain dependent LFP frequency spectra. Provided is the detailed map of LFP frequency spectra produced by LCM using different excitatory and inhibitory gains. The red lines show the frequency spectra of stimulated LFPs, while the black lines depict that of spontaneous LFPs. (TIF) [file pcbi.1002733.s001.tif]

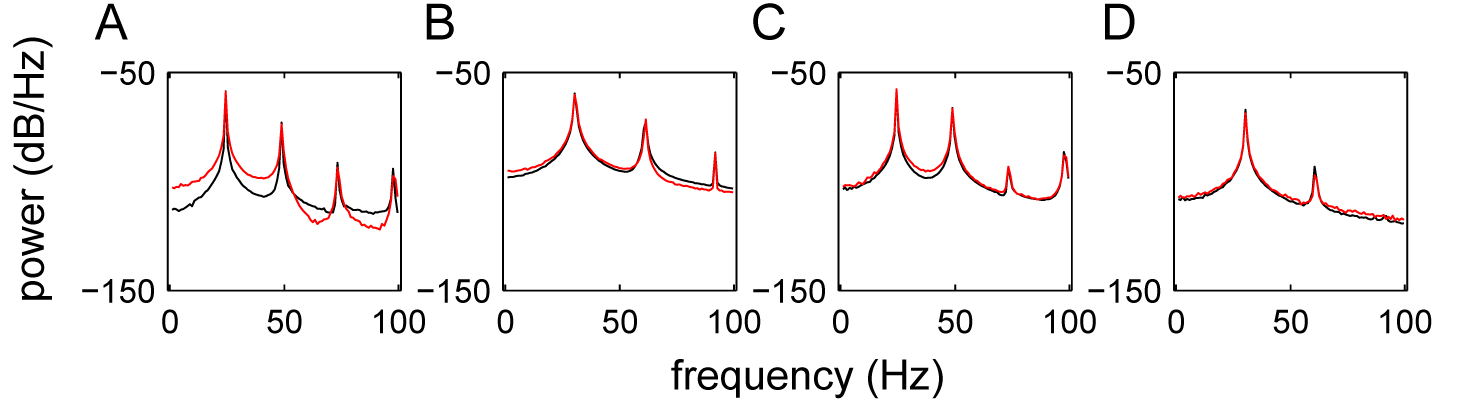

Supplement: Figure S2 — The shift of frequency peaks with different PSP time courses. Provided are LCM produced LFP frequency spectra while the peaks of EPSP time courses are (A) doubled and (B) decreased by half, and the peak of IPSP time course is (C) doubled and (D) decreased by half. The following parameter values were used: . (TIF) [file pcbi.1002733.s002.tif]
