# Supplementary material for: The Laminar Cortex Model: A New Continuum Cortex Model Incorporating Laminar Architecture
Source: PLoS Comput Biol. 2012 Oct 18;8(10):e1002733. doi: 10.1371/journal.pcbi.1002733 (PMC3475685; doi:10.1371/journal.pcbi.1002733)
Supplement: Text S1 — The state equations of the laminar cortex model. (DOC) [file pcbi.1002733.s003.doc]

# Text S1: The state equations of the laminar cortex model

The laminar cortex model (LCM) uses a simplified version of the continuum cortex model (CCM) to simulate neuronal processes . The equations used in the LCM, as listed here, are variations of the equations used in the continuum cortex model .

## Spike propagation

Spikes are initialized in the axon hillock of the soma and delivered through the axon tree to the target neurons . In the CCM, Wright et al. employed a mean field approximation assuming that spike propagation is isotropic and cortical synapses are distributed evenly. With the mean filed approximation, the amount of spikes received by a synapse in a unit time is

The integral is taken over the cortical layer. $Q_{p}(\mathbf{r}',t)$ is the average rate of the spike source at $\mathbf{r}'$. Subscript $p$ indicates the type of spikes (excitatory or inhibitory). $v_{p}$ is the spike spread speed. $f(\mathbf{r},\mathbf{r}')$ is the *spike propagator*. It represents the possibility for spikes generated at $\mathbf{r}'$ reaching synapses at $\mathbf{r}$. The spike propagators of single neurons depend on neurons’ morphology and physiology. However, under the mean field approximation, the spike propagator of neuron population is only a function of the distance $\left|\mathbf{r}-\mathbf{r}'\right|$. We use the following propagator in LCM

$$f(\mathbf{r},\mathbf{r}')=\exp\left(\frac{-\left|\mathbf{r}-\mathbf{r}'\right|^{2}}{2\gamma_{p}^{2}}\right)$$

where $\gamma_{p}$ is a parameter controlling the spike propagation range. We do not use a normalized spike propagator in the LCM, because spikes may be diminished or enhanced during their propagation in axons .

## Postsynaptic potential

The afferent spikes cause a postsynaptic membrane potential change in the target neuron. The postsynaptic potential (PSP) amplitude depends on afferent spike rate and the activation level of the target neuron. LCM uses the same formulation for the postsynaptic potential size as the CCM , stated as

where $g_{p}$ is the synaptic gain at resting membrane potential. $V_{p}^{\text{rev}}$ is the reversal membrane potential. Subscripts $p$ and $q$ indicate the type of the afferent spike and the target neuron. $\lambda^{[\text{rcpt}]}$ is a parameter describing spike adaptation of the receptor $\text{[rcpt]}$. Three types of neurotransmitter receptors are included in the LCM: *AMPA* receptor, *NMDA* receptor, and *GABA* receptor.

In the LCM, neurons receive spikes from four sources: LGN of the thalamus (), the current cortical layer (), other cortical layers (), and other cortical areas (). We assumed the spike rate from other cortical area () is a background noise, and used a small white noise to represent it. Spikes from LGN () are the simulation input, depending on stimulations used in the simulation. Spike from other cortical layers () is the summation of the spike from elements in the same horizontal position from all other layers

where the summation is taken over all layers except the current layer $i$; $\triangle r$ is the vertical distance between layer $i$ and $j$ ; $Q_{\text{ly}=j}(\mathbf{r},t-\triangle t)$ is the spike rate in cortical layer $j$ at the time of $t-\triangle t$; $\triangle t=\triangle r/v_{\text{v}}$is the time delay of spikes from layer $j$ to layer $i$.

The total membrane potential change is the summation of the membrane potential changes caused by all afferent spikes

$$M_{qp}^{\text{[rcpt]}}=N_{\text{ic, qp}}M_{\text{ic, p}}+N_{\text{cc, qp}}M_{\text{cc, p}}+N_{\text{th, qp}}M_{\text{th, p}}+\sum_{\text{other ly}} N_{\text{ly, qp}}M_{\text{ly, p}}$$

where $N_{qp}$ is the average number of neuron synapses between the two neuron groups, subscripts $\text{ic}$, $\text{ly}$, $\text{cc}$ and $\text{th}$ indicate the synapses is from the current cortical layer, other cortical layers, other cortical areas, and thalamus, respectively.

In CCM, the time course of PSP consists of the convolution of three different functions. In order to simplified the simulation, we used to a gamma function to represent them

$$R^{\text{[rcpt]}}(V_{q},t)=\left\{\begin{matrix} \frac{\alpha^{n}}{\Gamma(n)}(t-\tau^{\text{[rcpt]}})^{n-1}\exp\left[-\alpha(t-\tau^{\text{[rcpt]}})\right] & \text{when }t>\tau^{[\text{rcpt}]} \\ 0 & \text{otherwise} \end{matrix}\right.$$

where $\alpha$ and $n$ are parameters controlling the shape of PSP time course; $\tau^{[\text{rcpt}]}$ is the synaptic delay. The values of $\alpha$ and $n$ also depend on the membrane potential $V_{q}$, because the PSP time course varies with the membrane potential of the target neuron . LCM uses the following linear approximation

$\alpha(V_{\text{q}})=\alpha_{0}+k(V_{\text{q}}-V_{\text{q}}^{\text{[0]}})$,

$n(V_{\text{q}})=\frac{\alpha}{\alpha_{0}}(n_{0}-1)+1$.

Equations and ensure that the peaks of the time courses keep unchanged while their standard deviations vary with the membrane potential $V_{q}$linearly.

## Soma membrane potential aggregation

Under mean field approximation, the final neuronal membrane potential is

where denotes convolution in time.

## Spike generation

Because neurons generate spikes in a “all-or-none” way, the spike generation function for individual neurons is close to a step function. The activation thresholds usually vary among neurons. If we assume the thresholds are distributed normally in a large neuron group, then the mean firing rate of a neuron population is

$$Q_{q}\left(t\right)=\frac{Q_{q}^{\text{max}}}{1+\exp\left[-\pi(V_{q}-\theta_{q})/(\sqrt{3}\sigma_{q})\right]}$$

where $\theta_{q}\text{ }$and $\sigma_{q}$ are the mean and standard deviation of the thresholds.

## Source code

We provided the source code of the model in <http://www.uq.edu.au/~uqjdu2/> for the sake of completeness. We provide no guarantee or support for the program.

## References

1. Wright JJ (2009) Generation and control of cortical gamma: findings from simulation at two scales. Neural Netw 22: 373-384.

2. Wright JJ, Rennie CJ, Lees GJ, Robinson PA, Bourke PD, et al. (2003) Simulated electrocortical activity at microscopic, mesoscopic, and global scales. Neuropsychopharmacology 28 Suppl 1: S80-93.

3. Robinson PA, Rennie CJ, Wright JJ (1997) Propagation and stability of waves of electrical activity in the cerebral cortex. Phys Rev E Stat Nonlin Soft Matter Phys 56: 826-840.

4. Rennie CJ, Wright JJ, Robinson PA (2000) Mechanisms of cortical electrical activity and emergence of gamma rhythm. J Theor Biol 205: 17-35.

5. Kandel ER, Schwartz JH, Jessell TM (2000) Principles of neural science. New York: McGraw-Hill, Health Professions Division. xli, 1414 p. p.

6. Waxman SG, Kocsis JD, Stys PK (1995) The axon : structure, function, and pathophysiology. New York: Oxford University Press. xv, 692 p., 692 p. of plates p.

7. Thomson AM (1997) Activity-dependent properties of synaptic transmission at two classes of connections made by rat neocortical pyramidal axons in vitro. J Physiol 502: 131-147.

8. Thomson AM, West DC, Hahn J, Deuchars J (1996) Single axon IPSPs elicited in pyramidal cells by three classes of interneurones in slices of rat neocortex. J Physiol 496: 81-102.
