# Supplementary material for: The Laminar Cortex Model: A New Continuum Cortex Model Incorporating Laminar Architecture
Source: PLoS Comput Biol. 2012 Oct 18;8(10):e1002733. doi: 10.1371/journal.pcbi.1002733 (PMC3475685; doi:10.1371/journal.pcbi.1002733)
Supplement: Text S2 — The meanings and values of the parameters used in the laminar cortex model. (DOC) [file pcbi.1002733.s004.doc]

# Text S2: The parameters of the laminar cortex model

There are approximately 150 parameters in the laminar cortex model (LCM), most of which have been estimated from experimental data. We listed their meanings and values in the following.

## Laminar synaptic connection map

The LCM uses a quantitative map to define the synaptic connection between cortical layers. The connection map is derived from , in which the authors made three-dimensional reconstructions of 39 single neurons and thalamic afferents *in vivo* in cat primary visual cortex, and counted the synapses formed between neurons of different types (see Figure 7 and 8 in ). We calculated the average synapse numbers formed by excitatory and inhibitory neurons in each cortical layer from their data. The results are provided in Table S1 (data were acquired from ).

Table S1: Synapse numbers formed between neurons of different types. **Each row represents a postsynaptic neuron type, where e1 (i1) indicates excitatory (inhibitory) neuron of layer 1, and so on. Each column represents a presynaptic neuron type, where CC and X/Y indicate the presynaptic neuron is in other cortex areas and LGN, respectively.**

|  |  | Presynaptic neuron | | | | | | | | | | | |
| --- | --- | --- | --- | --- | --- | --- | --- | --- | --- | --- | --- | --- | --- |
|  |  | e1 | i1 | e2/3 | i2/3 | e4 | i4 | e5 | i5 | e6 | i6 | CC | X/Y |
| Postsynaptic neuron | e1 | 907 | 1600 | 907 | 160 |  |  |  |  |  |  | 7752 | 408 |
|  | i1 | 73 | 898 | 560 | 151 | 9 |  | 9 |  |  |  | 7191 |  |
|  | e2/3 |  | 133 | 3557 | 799 | 883 | 46 | 431 |  | 133 | 46 | 1019 | 54 |
|  | i2/3 |  | 54 | 1769 | 509 | 443 | 28 | 215 |  | 69 | 23 | 429 |  |
|  | e4 |  | 27 | 416 | 79 | 1073 | 488 | 82 |  | 1684 | 305 | 1507 | 169 |
|  | i4 |  |  | 168 | 39 | 635 | 357 | 35 |  | 1024 | 182 | 829 | 54 |
|  | e5 |  | 138 | 2526 | 168 | 756 | 71 | 620 | 85 | 360 | 547 | 1510 | 1692 |
|  | i5 |  |  | 1356 | 75 | 382 | 33 | 376 | 66 | 128 | 340 | 227 | 3 |
|  | e6 |  | 2 | 646 | 44 | 554 | 111 | 330 | 24 | 1100 | 784 | 2602 | 188 |
|  | i6 |  |  | 81 | 6 | 93 | 3 | 161 | 13 | 464 | 496 | 1887 | 19 |

There are a large number of unassigned symmetric and asymmetric synapses in the data of , because some neuron types, such as the spiny neuron in layer I and smooth neuron in all layers, are not included in their estimations. Following the suggestion of the authors , we assumed that: 1) The unsigned symmetric synapses are from the smooth neurons of the layer where the synapses are formed ; 2) 5% of unsigned asymmetric synapses come from the unspecific nuclei of thalamus ; 3) 95% of unsigned asymmetric synapses come from other cortical area . Since no information about excitatory neuron of layer I is provided, we further assume that: 4) 10% of unsigned asymmetric synapses on excitatory neurons of layer I are from excitatory neurons of the same layer. 5) 0.03% of unsigned asymmetric synapses on inhibitory neurons of layer I are from excitatory neurons of the same layer. Modification has been made to data in Table S1.

Table S2 lists the values of cortical depth, ratios of the number of neurons used in LCM. They are estimated from physiological experiment data .

**Table S2. Cortical depth and neuron numbers of each** layer

| Cortical layer | Cortical depth (mm) | Percentage of neurons in the cortex (%) | Percentage of excitatory neurons in the layer (%) |
| --- | --- | --- | --- |
| LI | 0.123 | 1.6 | 3 |
| LII/III | 0.526 | 33.8 | 78 |
| LIV | 1.133 | 34.9 | 80 |
| LV | 1.568 | 7.6 | 82 |
| LVI | 1.816 | 22.1 | 83 |

## Neuronal physiology parameters

LCM uses the same neuronal physiology parameters as CCM , given in Table S3.

**Table S3: Parameters for neuron electrophysiological property**

| Parameter | Meaning | Value |
| --- | --- | --- |
| $\text{Q}_{p}^{\text{max}}$ | Maximum firing rate of neuron | $\text{Q}_{\text{e}}^{\text{max}}=100$ spikes/sec $\text{Q}_{\text{i}}^{\text{max}}=200$ spikes/sec |
| $V_{p}^{\text{rev}}$ | Neuron reversal potential | $V_{\text{i}}^{\text{rev}}=-70\text{ mV}$ |
| $\text{V}_{p}^{[0]}$ | Neuron resting membrane potential | -64 mV |
| $\theta_{q}$ | Mean membrane potential when half of neurons are full firing | -35 mV |
| $\sigma_{q}$ | Standard deviation of neuron firing probability | Excitatory: 14.5 mV  Inhibitory: 12 mV |

## Postsynaptic time course parameters

The postsynaptic potential time course parameters were estimated from . Since the reported values of PSP time cover a large range, we chose the middle parameter value if a value range was provided or the average when multiple values had been reported.

**Table S4: Parameters for synaptic transmission**

| Parameter | Meaning | Value |
| --- | --- | --- |
| $a_{0}^{\text{[rcpt]}}$ | PSP change onset time constant | $\text{[AMPA]}=138.8$ $\text{[NMDA]}=42.6$ $\text{[GABA]}=68.5$ |
| $n_{0}^{[\text{rcpt}]}$ | PSP change onset time constant | $\text{[AMPA]}=1.70$ $\text{[NMDA]}=2.1$ $\text{[GABA]}=1.55$ |
| $\tau^{\left[\text{rcpt}\right]}$ | Synaptic delay | $\text{[AMPA]}=0.38\text{ msec}$ $\text{[NMDA]}=0.38\text{ msec}$ $[\text{GABA}]=0.9\text{ msec}$ |
| $k^{[\text{rcpt}]}$ | Parameter of PSP time course dependence on soma membrane potential | $[\text{AMPA}]=-0.3\text{ mV}^{-1}$ $[\text{NMDA}]=-\text{0.2 mV}^{-1}$ $[\text{GABA}]=0.5\text{ mV}^{-1}$ |
| $\lambda^{[\text{rcpt}]}$ | Receptor spike adaption parameters | $[\text{AMPA]}=12\text{ msec}$ $[\text{NMDA}]=37\text{ msec}$ $[\text{GABA}]=50\text{ msec}$ |

## References

1. Binzegger T, Douglas RJ, Martin KA (2004) A quantitative map of the circuit of cat primary visual cortex. J Neurosci 24: 8441-8453.

2. Izhikevich EM, Edelman GM (2008) Large-scale model of mammalian thalamocortical systems. Proc Natl Acad Sci U S A 105: 3593-3598.

3. O'Kusky J, Colonnier M (1982) A laminar analysis of the number of neurons, glia, and synapses in the adult cortex (area 17) of adult macaque monkeys. J Comp Neurol 210: 278-290.

4. Wright JJ (2009) Generation and control of cortical gamma: findings from simulation at two scales. Neural Netw 22: 373-384.

5. Thomson AM (1997) Activity-dependent properties of synaptic transmission at two classes of connections made by rat neocortical pyramidal axons in vitro. J Physiol 502: 131-147.

6. Thomson AM, West DC, Hahn J, Deuchars J (1996) Single axon IPSPs elicited in pyramidal cells by three classes of interneurones in slices of rat neocortex. J Physiol 496: 81-102.
